# Supplementary material for: 3D convolutional neural networks uncover modality-specific brain-imaging predictors for Alzheimer’s disease sub-scores
Source: Brain Inform. 2024 Feb 4;11(1):5. doi: 10.1186/s40708-024-00218-x (PMC10838875; doi:10.1186/s40708-024-00218-x)
Supplement: Supplementary file 1 — Additional file 1: Fig. S1. Imaging preprocessing. A Schematic representation of the image processing pipeline. Example of white-stripe normalization for MRI images, with pixel intensity distributions of the reference MRI (grey lines), a successfully normalized MRI (black lines) and an abnormal MRI that fails white stripe normalization (red lines) from raw imaging B to normalized imaging (C). Fig. S2. Receiver Operating Characteristic (ROC) curves of classification models on the ADNI and RADC data sets. A–C MRI, FDG–PET, and AV45–PET-based CNN models with diagnostic extension applied to ADNI samples. D MRI-based CNN models with diagnostic extension applied to RADC samples. Random forest (black), K nearest neighbours (grey), and logistic regression (blue) were used for the diagnosis extension. Fig. S3. Feature importance scores of selected brain regions within MRI A–D and FDG–PET E–H in different diagnosis sub-groups. Table S1. Demographic information of RADC samples. Table S2. Random forest feature importance for predicting AD vs. nAD based on ADAS–Cog13 sub-scores. Table S3. Accuracy of CNN model extension for AD diagnosis. [file 40708_2024_218_MOESM1_ESM.docx]

**Additional file 1**

**Figures and Tables**


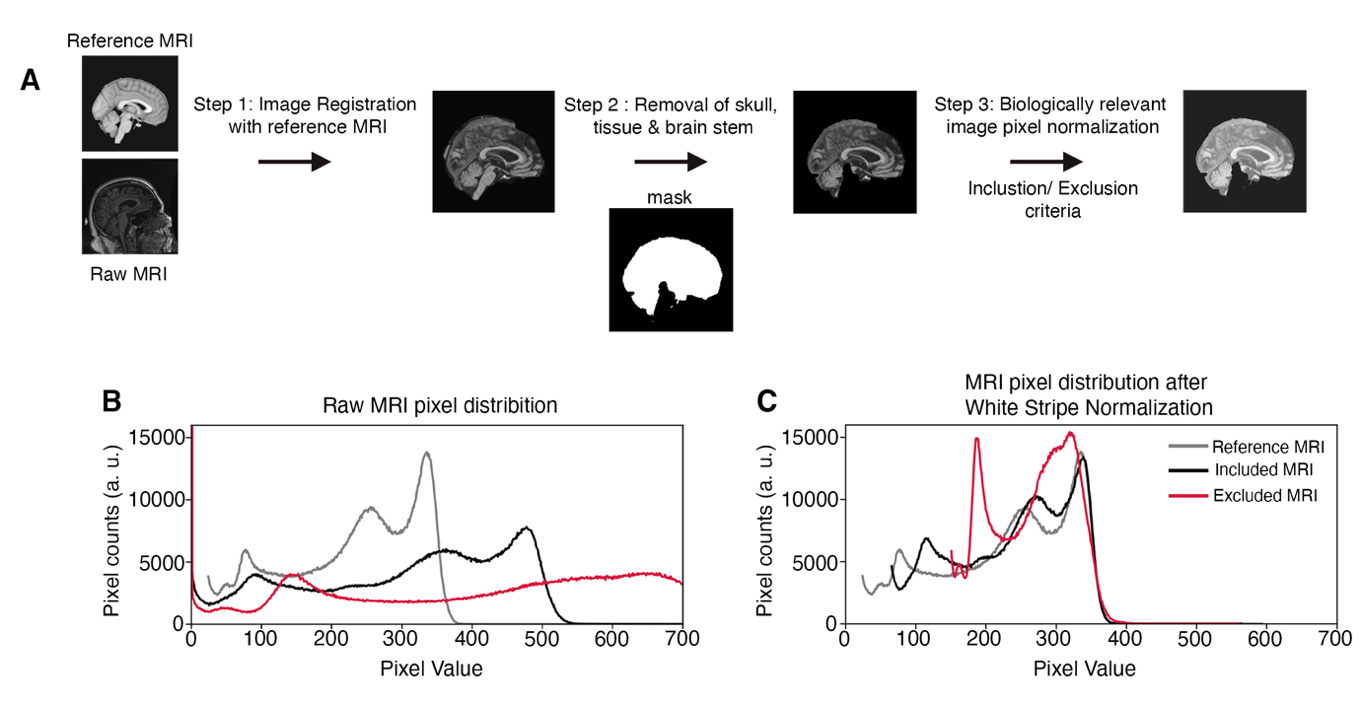


**Figure S1. Imaging preprocessing.** A) Schematic representation of the image processing pipeline. Example of white-stripe normalization for MRI images, with pixel intensity distributions of the reference MRI (grey lines), a successfully normalized MRI (black lines) and an abnormal MRI that fails white stripe normalization (red lines) from raw imaging (B) to normalized imaging (C).

**
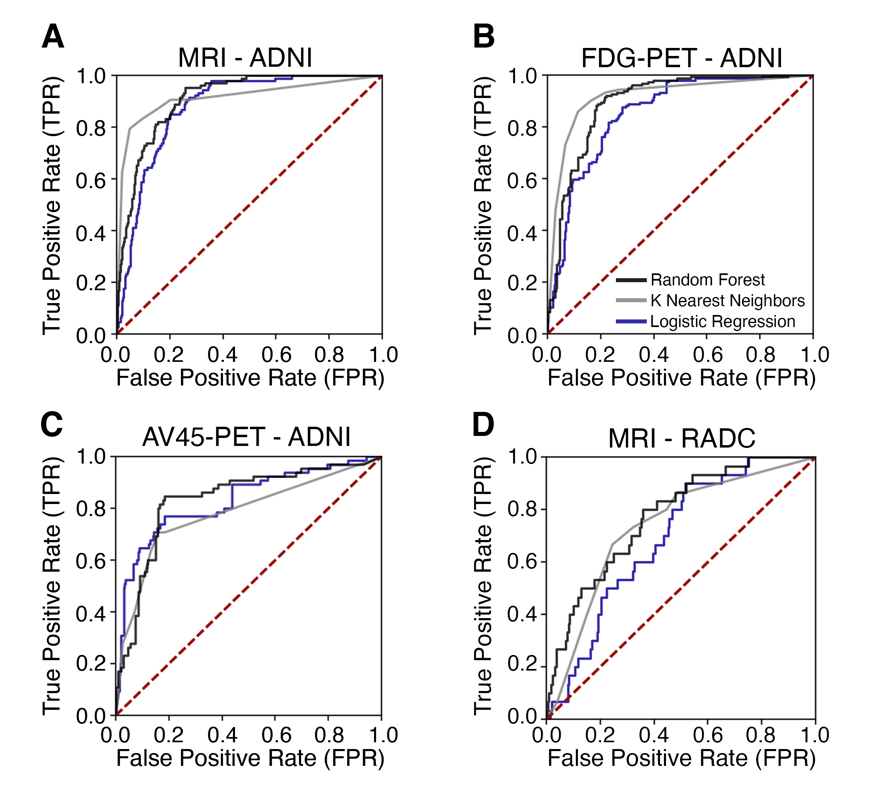
**

**Figure S2.** Receiver Operating Characteristic (ROC) curves of classification models on the ADNI and RADC datasets. A-C: MRI, FDG-PET, and AV45-PET based CNN models with diagnostic extension applied to ADNI samples. D: MRI based CNN models with diagnostic extension applied to RADC samples. Random forest (black), K nearest neighbours (grey), and logistic regression (blue) were used for the diagnosis extension.


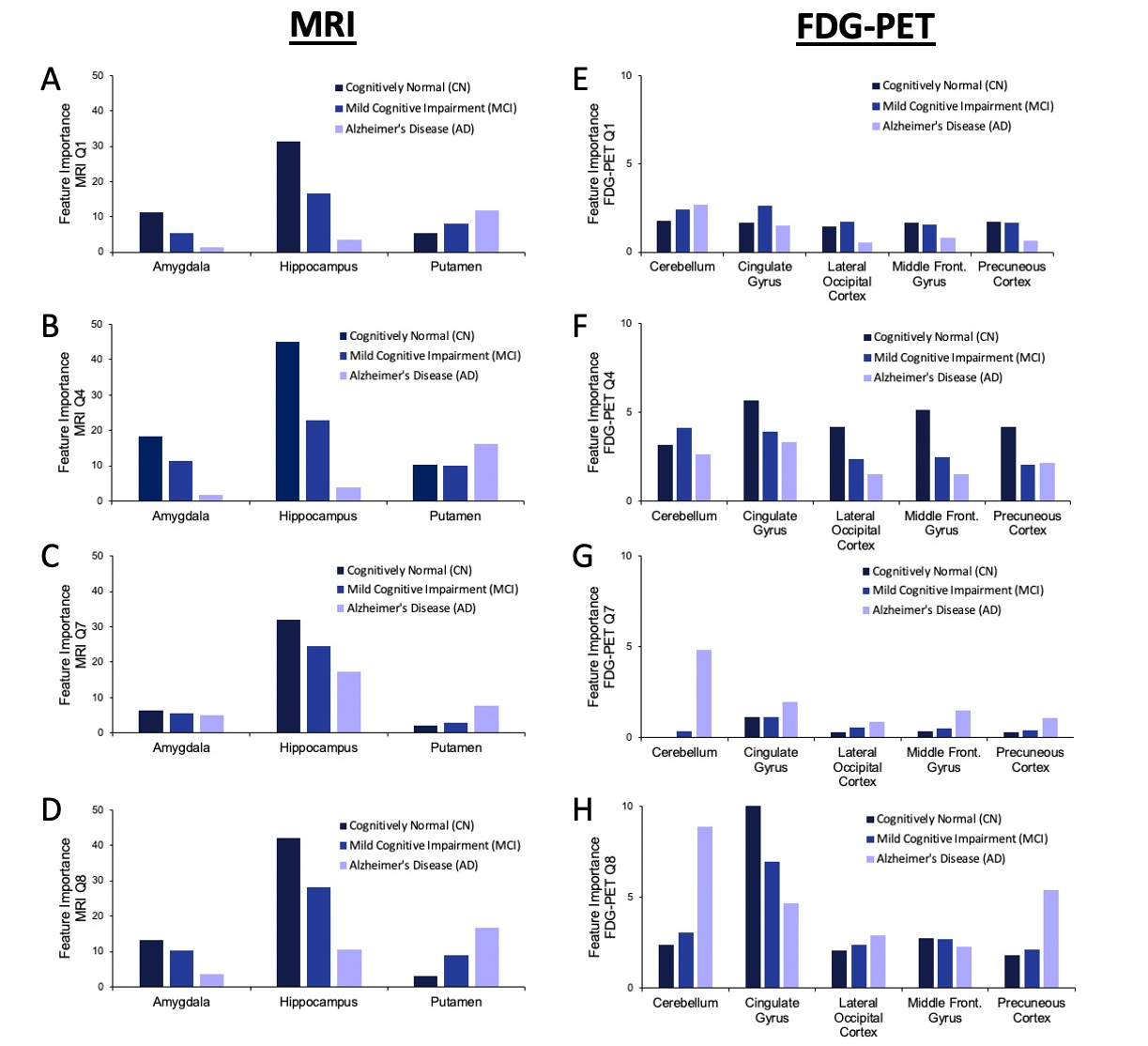


**Figure S3.** Feature importance scores of selected brain regions within MRI (A-D) and FDG-PET (E-H) in different diagnosis sub-groups.

**Table S1**. Demographic information of RADC samples.

|  |  | **Normal cognition** | **MCI** | **Alzheimer’s Disease** |
| --- | --- | --- | --- | --- |
|  |  | (n = 1641) | (n = 325) | (n = 30) |
|  |  | **Count (%)** | **Count (%)** | **Count (%)** |
| **Sex** | **F** | 1271 (77) | 246 (75) | 20 (66) |
|  | **M** | 370 (23) | 79 (24) | 28 (34) |
|  | | **Mean (stdev)** | **Mean (stdev)** | **Mean (stdev)** |
| **Age** | | 81.0 (7.3) | 85.0 (6.2) | 86.7 (7.6) |
| **Total MMSE** | | 28.6 (1.3) | 26.9 (2.1) | 23.0 (3.1) |

**Table S2.** Random forest feature importance for predicting AD vs. nAD based on ADAS-Cog13 sub-scores.

| ADAS-Cog sub-score | feature importance (%) |
| --- | --- |
| Q1 – Word recall | 16 |
| Q2 – Commands | 1 |
| Q3 – Constructional praxis | 1 |
| Q4 – Delayed word recall | 29 |
| Q5 – Naming objects/fingers | 2 |
| Q6 – Ideational praxis | 3 |
| Q7 – Orientation | 25 |
| Q8 – Word recognition | 11 |
| Q9 – Remembering test instructions | 1 |
| Q10 – Comprehension | 1 |
| Q11 – Word finding language ability | 2 |
| Q12 – Spoken language ability | 1 |
| Q13 – Number cancellation | 5 |

**Table S3.** Accuracy of CNN model extension for AD diagnosis.

| **Model** | **AUROC** |
| --- | --- |
| **MRI** | |
| Logistic regression | 0.78 |
| K-nearest neighbors | 0.89 |
| Random forest | 0.83 |
| **FDG-PET** | |
| Logistic regression | 0.85 |
| K-nearest neighbors | 0.9 |
| Random forest | 0.86 |
| **AV45-PET** | |
| Logistic regression | 0.61 |
| K-nearest neighbors | 0.84 |
| Random forest | 0.71 |
